# A Bioinspired Swimming and Walking Hydrogel Driven by Light‐Controlled Local Density

**DOI:** 10.1002/advs.201500084

**Published:** 2015-05-15

**Authors:** Lei Wang, Yang Liu, Yao Cheng, Xiuguo Cui, Huiqin Lian, Yongri Liang, Fei Chen, Hao Wang, Wenli Guo, Hangquan Li, Meifang Zhu, Hirotaka Ihara

**Affiliations:** ^1^ Beijing Key Lab of Special Elastomer Composite MaterialsCollege of Materials Science and EngineeringBeijing Institute of Petrochemical Technology19 North Qingyuan RoadBeijing102617P.R. China; ^2^ College of Materials Science and EngineeringBeijing University of Chemical TechnologyBeijing100029P.R. China; ^3^ State Key Lab for Modification of Chemical Fibers and Polymer MaterialsCollege of Materials Science and EngineeringDonghua University2999 Ren‐min RoadShanghai201620P.R. China; ^4^ Department of Applied Chemistry and BiochemistryKumamoto UniversityKumamoto860‐8555Japan

**Keywords:** actuator, bioinspired material, graphene, hydrogel, nanocomposite

## Abstract

**A hydrogel exhibits a real‐time depth‐controllable swimming motion** via light‐mediated modulation of local density to mimic the volume changes found in the bladders of fish. Moreover, other motions, e.g., rolling, somersaulting, and bipedal‐like walking, can also be realized by designing or combining gel shapes, and the location of light.

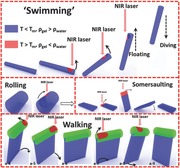

An actuator is a kind of device that can transform a certain energy source into motion, which has been applied in a lot of fields.^1^ Many types of motions have been realized by researchers, e.g., crawling,^2^ bending/folding,[[qv: 1b,3]] rolling,^4^ jumping,^5^ walking,[[qv: 3e,6]] diving–floating,^7^ microjeting in aqueous solutions,^8^ moving on water surface,^9^ etc. Among the motions, diving–floating, microjeting in aqueous solutions, and moving on water surface can be included in “swimming” motions.^7^, ^8^, ^9^, ^10^ Gao et al. first fabricated a cooperating device by combining a pH‐responsive surface with hydrogen peroxide‐responsive platinum on a nickel foam cube, which displays a diving–floating motion in H_2_O_2_ solution.[[qv: 7a]] Afterwards, researchers further fabricated thermosensitive diving–floating devices for matter transportation between three phases,[[qv: 7b]] or used diving–floating devices to convert chemical energy into electricity.[[qv: 7d]] However, a real swimming motion in nature should be depth‐controllable, and the reported “swimming” motions cannot attain this. Here, we show a hydrogel which exhibit a real‐time depth‐controllable swimming motion based on a new concept of light‐controlled local density inspired by fish swimming. The swimming depth of the gel can be instantly controlled by near infrared (NIR) laser; moreover, other motions, e.g., rolling, somersaulting, and “bipedal‐like” walking, can also be realized by designing or combining gel shapes, different gel compositions, and the location of NIR laser. Our results demonstrate that the velocity during swimming and other motions can also be modulated by NIR laser. We anticipate that the new concept of light‐controlled local density of materials will be a starting point for realizing complicated swimming motions and other various motions of actuators, which will be applied in mini‐robots in future. Furthermore, the actuators and mini‐robots based on this concept could be composites of polymers, ceramics, or even metals, and the environment for motions could be other transparent liquids, not limited to water.

As fishes swim, they modulate their buoyancy to control depth by changing the volume of swimming bladders, i.e., varying the local density of their bodies. Inspired by swimming bladders of fish, the concept of designing the swimming gel is illustrated in **Scheme** **1**. The material is made from three ingredients: monomer A (*ρ*_A_ < *ρ*_water_), monomer B (*ρ*_B_ > *ρ*_water_), and NIR absorbent C (small amount). After copolymerization, the density of the obtained material is above *ρ*_water_ by modulating the ratio of A and B, and the material contains a crystal phase of A. If temperature is higher than the melting point (*T*_m_) of A, crystals melt, decreasing *ρ*. As soon as the decreasing *ρ* is below *ρ*_water_, the material will float to water surface. Because the NIR laser absorbent can absorb laser energy, increasing temperature, the material will exhibit not only a thermosensitive floating/diving motion but also a light‐controlled swimming, rolling, somersaulting, and walking motions by modulating NIR laser location and time.

**Scheme 1:**
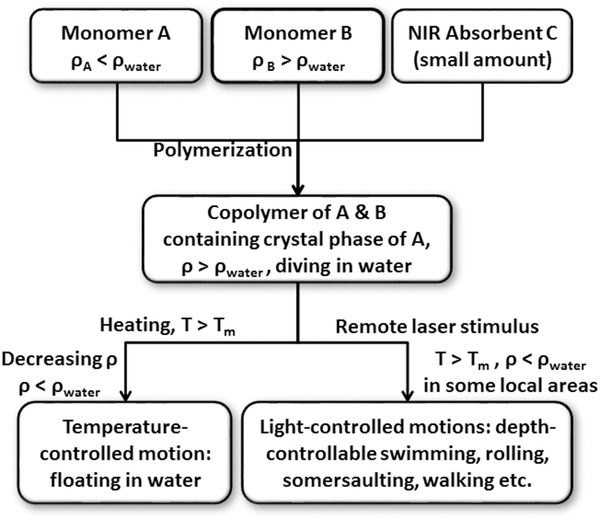
The concept of a light‐controlled local density of materials for swimming, rolling, somersaulting, and walking motions.

Here, stearyl acrylate (SA) is chosen as mono­mer A, whose density is 0.8 g cm^−3^;^11^ methacrylic acid (MA) is monomer B, whose density is 1.0153 g cm^−3^ (20 °C);^11^ reduced graphene oxide (RGO) is used as NIR absorbent, whose concentration is fixed on 0.6 wt%. The density of the copolymer can be controlled above *ρ*_water_ at room temperature by modulating compositions. The obtained copolymer of SA and MA contains a crystal phase resulting from the crystallization of the long side chains of SA. The long alkane side chains are arranged in a dense highly ordered microscopic structure in crystal phase, while they are arranged in a loose nonordered microscopic structure in melting state (Figure S1, Supporting Information). Thus, the density of crystal phase is higher than that of melting state, and we can change the density of the hydrogels by controlling the melting–crystalling phase transition. When temperature increases above the melting point, the crystal phase melts, decreasing *ρ*_gel_ (i.e., S53M47G0.6, where S, M, G stand for SA, MA, and RGO, respectively, 53, 47, 0.6 stand for mol% of SA, mol% of MA, and weight percent of RGO, respectively) below *ρ*_water_. (**Figure** **1**a). Then, the gel floats up. As temperature decreases, crystals form in the gel, increasing *ρ*_gel_ above *ρ*_water_, and the gel dives in water. As shown in Figure 1b, S53M47G0.6 (left one) exhibits a thermosensitive floating/diving motion. On the contrary, S82M18G0.6 (where S, M, G stand for SA, MA, and RGO, respectively, 82, 18, 0.6 stand for mol% of SA, mol% of MA, and weight percent of RGO, respectively) floats on water surface regardless the temperature, which means the *ρ*_gel_ of S82M18G0.6 is always below *ρ*_water_.

**Figure 1:**
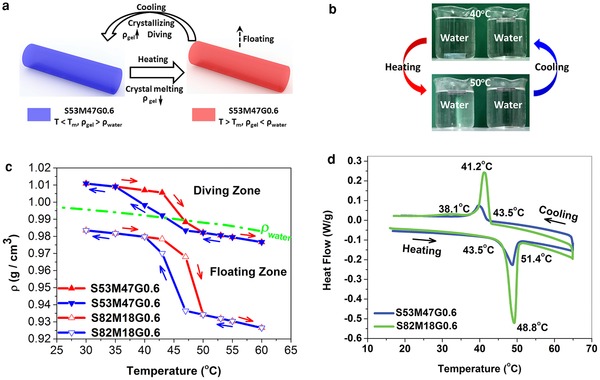
a) Mechanism of thermosensitive floating/diving motion of gels. b) Photographs of thermosensitive floating/diving motion (left: S53M47G0.6, right: S82M18G0.6). c) Density dependence of gels on temperature (red curves: heating process, blue curves: cooling process). d) DSC curves of S53M47G0.6 and S82M18G0.6 gels. S*x*M*y*G*z*: where S, M, G stand for SA (H_2_C=CHCO_2_(CH_2_)_17_CH_3_), MA (H_2_C=C(CH_3_)COOH), and RGO, respectively, *x, y, z* stand for mol% of SA, mol% of MA, and weight percent of RGO, respectively.

The density dependence of S53M47G0.6 and S82M18G0.6 on temperature is shown in Figure 1c. The whole area can be divided into two zones by a green line of *ρ*_water_. In the zone above this line, *ρ*_gel_ > *ρ*_water_, gels dive in water, so the zone is called “diving zone.” By contrast, the zone below the line is called “floating zone” where *ρ*_gel_ < *ρ*_water_ and gels float on water surface. First, the *ρ*_gel_ of S53M47G0.6 is in the “diving zone” at 30 °C. As temperature increases, *ρ*_gel_ slowly decreases until 43 °C. Then, *ρ*_gel_ decreases abruptly between 43 and 50 °C. At 50 °C, the *ρ*_gel_ of S53M47G0.6 arrives in the “floating zone,” and the gel floats. Similarly, during the cooling process from 60 to 30 °C, an abrupt increase in *ρ*_gel_ occurs between 47 and 35 °C. The *ρ*_gel_ of S53M47G0.6 returns to the “diving zone” at 43 °C, and the gel dives. S53M47G0.6 and S82M18G0.6 both exhibit an abrupt change and an obvious hysteresis cycle in *ρ*_gel_ between 35 and 50 °C. The abrupt change and obvious hysteresis of *ρ*_gel_ agree with the melting peaks (43.5–51.4 °C) and the crystallizing peaks (38.1–43.5 °C) of differential scanning calorimeter (DSC) curves in Figure 1d. This result confirms that the melting/crystallizing transition leads to the density change, making a thermosensitive floating/diving motion. It is apparent that the thermosensitive floating/diving motion is not a real‐time depth‐controllable motion, because changing external environment temperature is time‐consuming, not a real‐time method.

Light is a real‐time stimulus, so it is possible to instantly control the depth of floating/diving motion by modulating irradiating time. Based on the thermosensitive floating/diving motion and the introduced NIR absorbent (i.e., RGO), an S53M47G0.6 gel rod can be a light‐controlled swimming gel. When a NIR laser irradiates on the right bottom of the rod, the bottom region absorbs laser, where temperature increases above *T*_m_ and crystals melt, decreasing the local density; then the right bottom drives the whole rod floating up to water surface. The gel floats from the bottom of container to water surface, covering a depth of 11 cm during 13 s (from 2 to 14 s) by continuously applying a NIR laser on it; and it dives from water surface to the bottom of container during 9 s (from 17 to 26 s) after removing the NIR laser (**Figure** **2**b‐1 and Movie 1, Supporting Information). The velocity changes from −1.39 cm s^−1^ (floating) to 1.63 cm s^−1^ (diving) during the floating/diving process (Figure 2b‐2).

**Figure 2:**
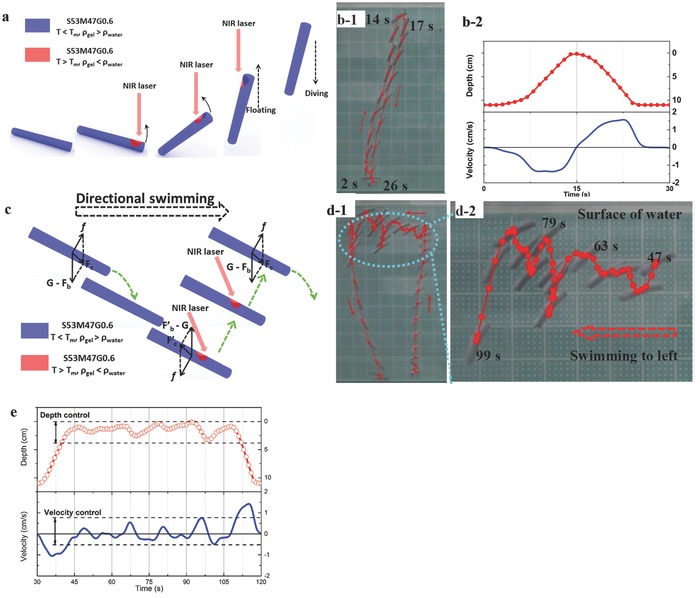
a) Mechanism of a light‐controlled diving/floating motion. b‐1) Photographs of a diving/floating motion, where a white square is 1 × 1 cm. b‐2) Depth versus time, and velocity versus time in the diving/floating motion. c) Mechanism of a depth‐controllable directional swimming motion. d‐1,d‐2) Photographs of the depth‐controllable directional swimming motion. e) Depth versus time, and velocity versus time in the depth‐controllable directional swimming motion.

Furthermore, the diving track of the gel rod is not completely vertical, because the diving track of the gel rod is related to its pose: an initial leaning pose results in a leaning diving track. The forces acting on the gel rod are shown in Figure 2c, where G, *F*_b_, *f*, and *F*_c_ stand for gravity, buoyancy, dynamic fluid obstruction, and composite force, respectively. As the gel rod is diving, the obstruction (*f*) pushes the gel rod forward horizontally, which explains the reason of the leaning diving track. If the NIR laser is applied to the gel rod to lift it during the diving process, the rod is anticipated to float up and move forward horizontally at the same time due to the inertia, illustrated in Figure 2c. The diving/floating cycle can be utilized to obtain a directional swimming motion in water. As shown in Figure 2d, the gel rod swims directionally to left between 47 and 99 s. Moreover, the depth and the floating/diving velocity of the gel rod are real‐time controlled by NIR laser (Figure 2e). The depth and the vertical velocity are modulated between 0 and 3.3 cm, and −0.53 and 0.76 cm s^−1^ during 40 and 110 s, respectively. The whole swimming motion is shown in Movie 1, Supporting Information.

Inspired by the swimming motion, a rolling motion can be realized on a short thick gel rod (**Figure** **3**a). If a NIR laser irradiates on one side of the rod, crystals melt in this area, decreasing *ρ*_gel_ in this local part. Then, the composite force of buoyancy and gravity give rise to a torque, rolling the short rod towards the other side. Changing the irradiated location of the NIR laser on the hydrogel rod, the rolling direction and velocity can be controlled (Figure 3b and Movie 2, Supporting Information). Moreover, somersaulting motion can also be realized on a rectangular gel plate (Figure 3c). If a NIR laser irradiates on the left bottom of the plate, crystals in this area melt, decreasing *ρ*_gel_ in the local part, and rotating the plate about the right bottom. When the plate rotates nearly to 90°, the NIR laser is removed, and the plate rotates across the highest point under inertia effect, completing a somersaulting action (Figure 3d and Movie 3, Supporting Information).

**Figure 3:**
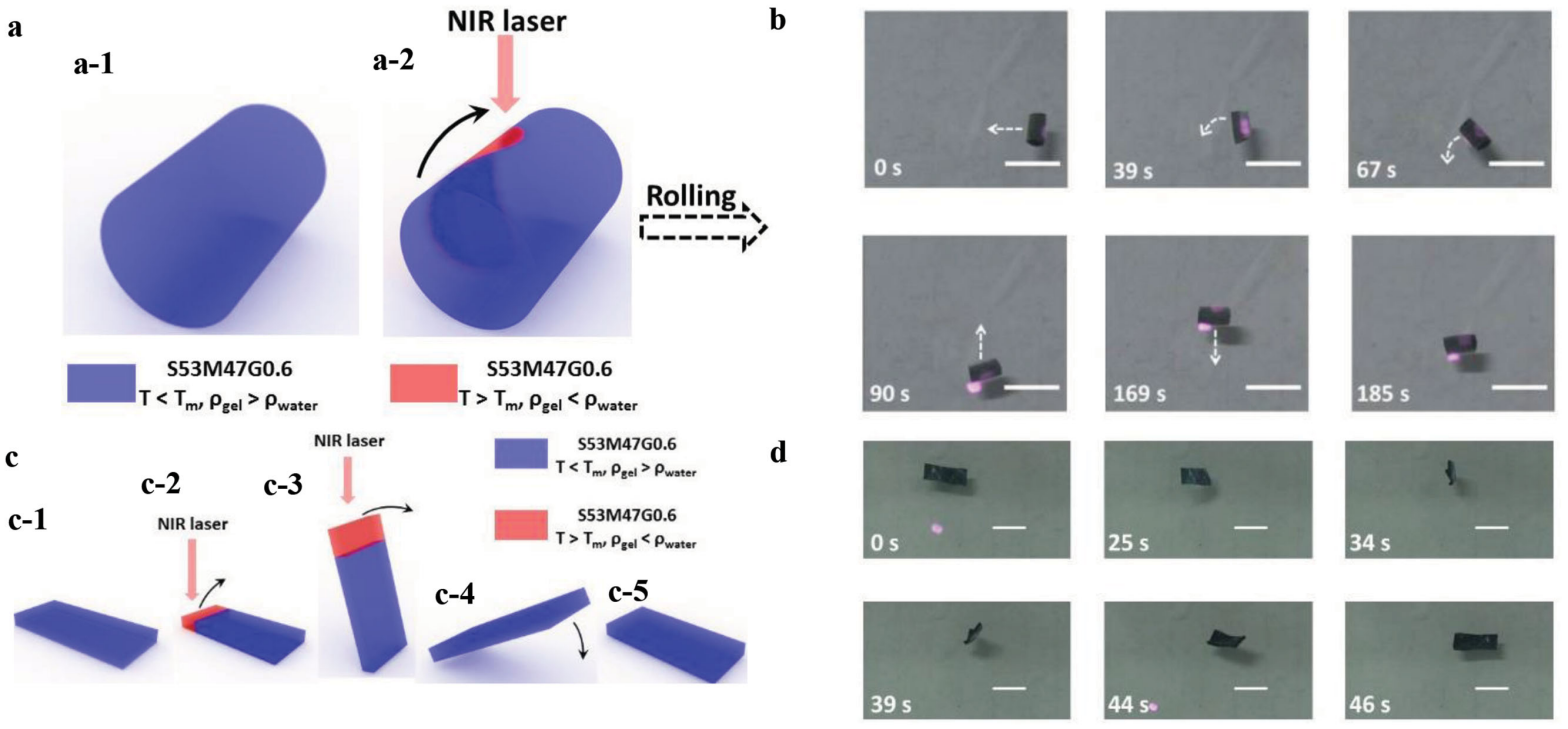
a) Mechanism of a light‐controlled rolling motion. b) Photographs of a light‐controlled rolling motion, white bar: 1 cm. c) Mechanism of a light‐controlled somersaulting motion. d) Photographs of a light‐controlled somersaulting motion.

Furthermore, a “bipedal‐like” walking gel minirobot can be obtained by combining S82M18G0.6 and S53M47G0.6 (**Figure** **4**a). First, both S82M18G0.6 and S53M47G0.6 are hard and crisp at room temperature. Then, S53M47G0.6 is kept at room temperature, while S82M18G0.6 is heated to 70 °C, above the *T*_m_ of crystal phase, becoming soft and sticky. Here, the hot sticky S82M18G0.6 and the cool hard S53M47G0.6 are pushed together until the S80MA20G0.6 is cooled to room temperature. Then, S82M18G0.6 and S53M47G0.6 stick together, forming a “composite” gel mini‐robot (CGM). The S82M18G0.6 part of the CGM is called “S82‐part,” and the other part is called “S53‐part.” The center of rod part in the CGM is above the symmetric axis (median of the rectangular part), while the gravity center of S53‐part is right on the median of rectangle, shown in Figure 4a. The CGM can exhibit “standing” and “walking” motions, irradiated by a NIR laser (Figure 4b,c). First, the NIR laser irradiates on the center of the S82 part, and the buoyancy of the S82‐part increases, rotating the CGM about the left bottom of S53‐part. This makes the CGM a “standing” pose, but slightly leaning ahead, because the torque induced by the gravity of S53‐part is equal to the torque induced by the buoyancy of S82‐part at this position. When the NIR laser irradiates on the “left shoulder” of CGM, the CGM rotates upwards about the “right leg” (Figure 4b‐4,b‐5); and the “left shoulder” falls to the ground, returning to the “leaning” pose, as the NIR laser removes (Figure 4b‐6). In the laser irradiating/removing cycle, the CGM steps the left leg. Similarly, the CGM steps another leg in the next cycle. Again and again, the CGM walks forward like a bipedal man, shown in Figure 4c and Movie 4, Supporting Information. The position of “left shoulder” or “right shoulder” exhibits a stepwise movement (Figure 4d), according to each step‐length.

**Figure 4:**
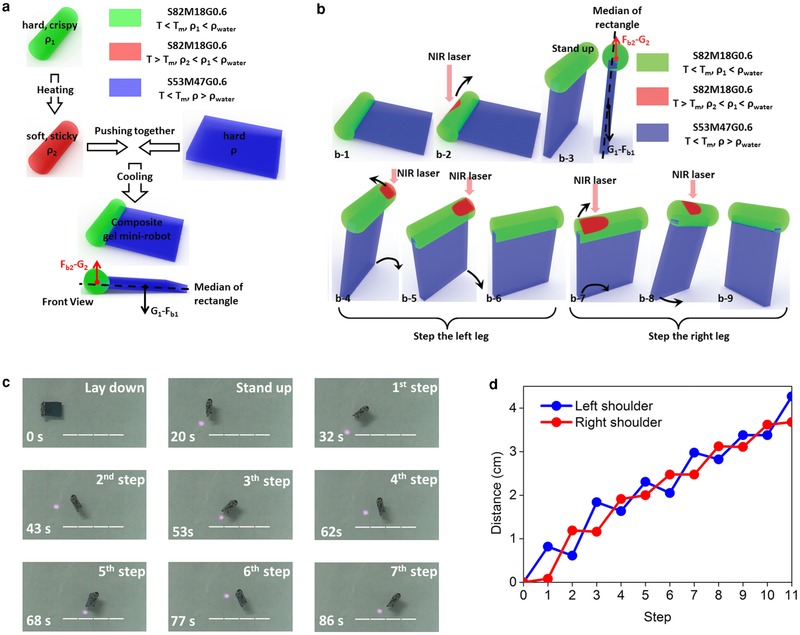
a) A scheme for a composite gel mini‐robot made of S53M47G0.6 and S82M18G0.6. b) Mechanism for a light‐controlled “bipedal‐like” walking motion. c) Photographs of the light‐controlled “bipedal‐like” walking motion, white bar: 1 cm. d) Moving distance of left shoulder and right shoulder on every step.

In conclusion, inspired by swimming bladders of fish, we have successfully prepared a real‐time depth‐controllable swimming gel. Based on the swimming motion, we have further realized rolling, somersaulting, and bipedal‐like walking motions by modulating and combining gel shapes, different gel compositions, and the location of NIR laser. Moreover, this concept of light‐controlled local density for various underwater motions could be used to realize motions not only in water but in other liquids as well, and the materials obtained on the concept may expand to plastics, rubbers, metals, or ceramics. This concept is believed to be widely applied in actuators and mini‐robots in future.

## Experimental Section

*Materials*: Stearyl acrylate, methacrylic acid, sodium ascorbate, *N*,*N*′‐methylenebisacrylamide (Bis), azobisisobutyronitrile (AIBN) were purchased from J&K Scientific Co. AIBN was purified by recrystallization before use. The ethanol dispersion of graphene oxide (GO) was offered by Beijing Carbon Century Technology Company.

*Synthesis*: S*x*M*y*G0.6 gels with different compositions were prepared by copolymerization (F = 0.53 and 0.82, F is defined as the mole fraction of SA in the total monomer). The total monomer concentration in an ethanol dispersion of GO (0.6 wt%) was kept at 4 mol kg^−1^ in the presence of 0.18 mol kg^−1^ AIBN and 3.9 × 10^−2^ mol kg^−1^ Bis. Polymerization was carried out at 50 °C in glass tubes for 12 h to form gels. After polymerization, the gel was immersed in a large amount of ethanol for more than 3 d to remove the monomers and then soaked in water for a week. Later, the gels were immersed in an aqueous solution of 1 wt% sodium ascorbate for a week at room temperature and following 1 d at 70 °C to reduce GO to RGO. Finally, the gels were immersed in deionized water again for a week to remove remaining sodium ascorbate. In this paper, the obtained gels are expressed as S*x*M*y*G0.6 where S, M, and G stand for SA, MA, and RGO, respectively, and *x, y*, and 0.6 stand for 100×SA/(SA+MA) (mol/mol), 100×MA/(SA+MA) (mol/mol), and 100×GO/ethanol (w/w), respectively.

*Density Measurement*: An equilibrium swollen gel rod (*φ* = 1.8 mm, length = 12 mm) was immersed in water at a certain temperatures for 30 min, then it was taken out and put into two alcohol/water solutions at the same temperature for 10 s. The two alcohol/water solutions have approaching compositions and densities. The floating or diving states of the gel rod in the mixture solutions were recorded. The density of the mixture solution, where the gel floats, is called *ρ*_f_; and the density of another mixture solution, where the gel dives, is called *ρ*_d_. The density of the gel, *ρ*_gel_, is equal to the average of *ρ*_d_ and *ρ*_f_. The gel rod was taken back into water again, and the temperature was increased to another value and was kept for 30 min. Then, the above procedure was carried on at every certain temperature until 60 °C. After density measurement at 60 °C, the water and solutions were cooled along the reverse heating path. During the cooling process, the density measurement was the same as that in heating process. Water and solutions were heated in water‐bath, and the bottoms of containers did not directly touch the heating plate. The details are illustrated in Figure S3 and Tables S1–S5, Supporting Information.

*Laser Actuation*: The swimming motion was characterized by immersing an equilibrium swollen gel rod (*φ* = 1.8 mm, length = 12 mm) in water. The gel rod was then irradiated by an 808 nm near‐IR laser (2 W output, Leimai Co.). Methods to characterize rolling, somersaulting, and walking motions are the same as that used in the swimming motion, except using a short thick gel rod (*φ* = 3.4 mm, length = 5 mm) a rectangular gel plate (0.9 × 4.8 × 12.7 mm), a gel rod of S82M18G0.6 (*φ* = 3.1 mm, length = 10 mm) and a gel plate (0.9 × 10 × 12 mm), respectively. All samples were equilibrium swollen hydrogels. Swimming and walking distance/velocity were determined by measuring the location change of central point of the gel rod using individual video frames. The tracing way used to irradiate different positions of the hydrogel was a manual control. The manipulator manually controlled the irradiated positions.

*Differential Scanning Calorimeter (DSC) (Q20, TA Instrument Company)*: 10 mg samples were sealed in an aluminum pan. The heating/cooling cycle is as follows under N_2_ atmosphere: the samples were heated at a rate of 1 °C min^−1^ from 15 to 65 °C, and were isothermally kept at 65 °C for 10 min, then they were cooled at a rate of −1 °C min^−1^ from 65 to 15 °C.

*Transmission Electron Microscopy (TEM) (H‐7650B, Hitachi Company)*: Hydrogel samples were dried at 70 °C in air. Then the dried gels were cut to 50 nm thin films for TEM. The accelerating voltage is 100 kV.

## Supporting information

As a service to our authors and readers, this journal provides supporting information supplied by the authors. Such materials are peer reviewed and may be re‐organized for online delivery, but are not copy‐edited or typeset. Technical support issues arising from supporting information (other than missing files) should be addressed to the authors.

Supplementary

Supplementary

Supplementary

Supplementary

Supplementary
