# Supplementary material for: A Bioinspired Swimming and Walking Hydrogel Driven by Light‐Controlled Local Density
Source: Adv Sci (Weinh). 2015 May 15;2(6):1500084. doi: 10.1002/advs.201500084 (PMC5115409; doi:10.1002/advs.201500084)
Supplement: Supplementary file 1 — Supplementary [file ADVS-2-0a-s002.pdf]

Copyright WILEY-VCH Verlag GmbH & Co. KGaA, 69469 Weinheim, Germany, 2015.

ADVANCED SCIENCE
Open Access

Supporting Information

for *Adv. Sci.*, DOI: 10.1002/advs.201500084

A Bioinspired Swimming and Walking Hydrogel Driven by Light-Controlled Local Density

Lei Wang, Yang Liu, Yao Cheng, Xiuguo Cui, Huiqin Lian, Yongri Liang, Fei Chen, Hao Wang, Wenli Guo, Hangquan Li, Meifang Zhu, and Hirotaka Ihara*

Copyright WILEY-VCH Verlag GmbH & Co. KGaA, 69469 Weinheim, Germany, 2013.

Supporting Information

A bio-inspired swimming and walking hydrogel driven by light-controlled local density

Lei Wang, Yang Liu, Yao Cheng, Xiuguo Cui, Huiqin Lian, Yongri Liang, Fei Chen, Hao Wang, Wenli Guo, Hangquan Li, Meifang Zhu, and Hirotaka Ihara*

Figure S1. Mechanism for the density change of melting/crystallizing phase transition in copolymer of SA and MA.

Figure S2. TEM images of S53M47G0.6

Figure S3. Density measurement of gels at different temperatures

Table S1. Density of glycerol solutions (g/cm^3)

Table S2. Density of ethanol solutions (g/cm^3)

Table S3. Density of pure water (g/cm^3)

Table S4. Density of S53M47G0.6 at different temperatures

Table S5. Density of S82M18G0.6 at different temperatures

Supplementary Movie 1: A real-time depth-controllable directional swimming motion

Supplementary Movie 2: A light-controlled rolling motion

Supplementary Movie 3: A light-controlled somersaulting motion

Supplementary Movie 4: A bipedal-like walking motion

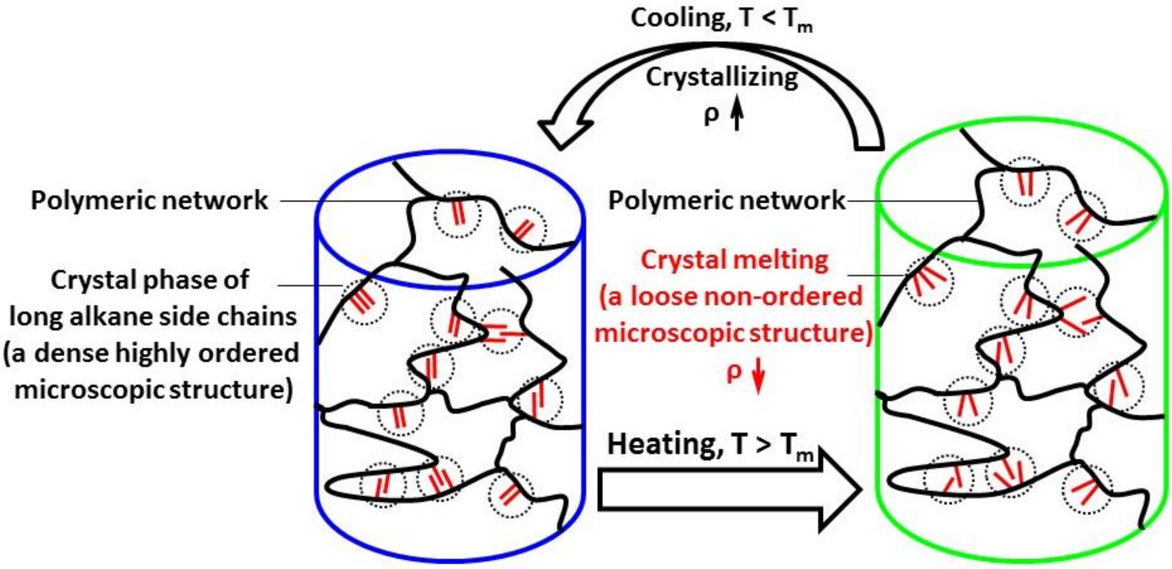

Figure S1. Mechanism for the density change of melting/crystallizing phase transition in copolymer of SA and MA.

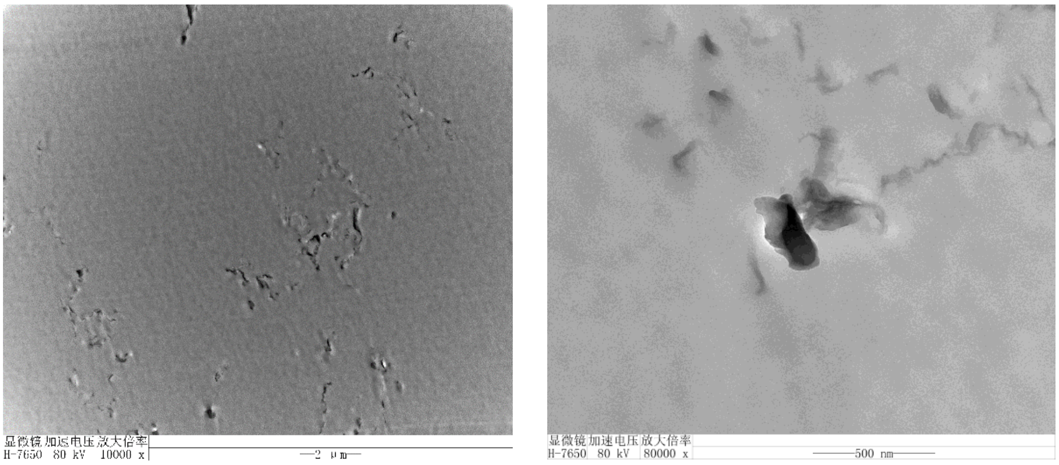

Figure S2. TEM images of S53M47G0.6

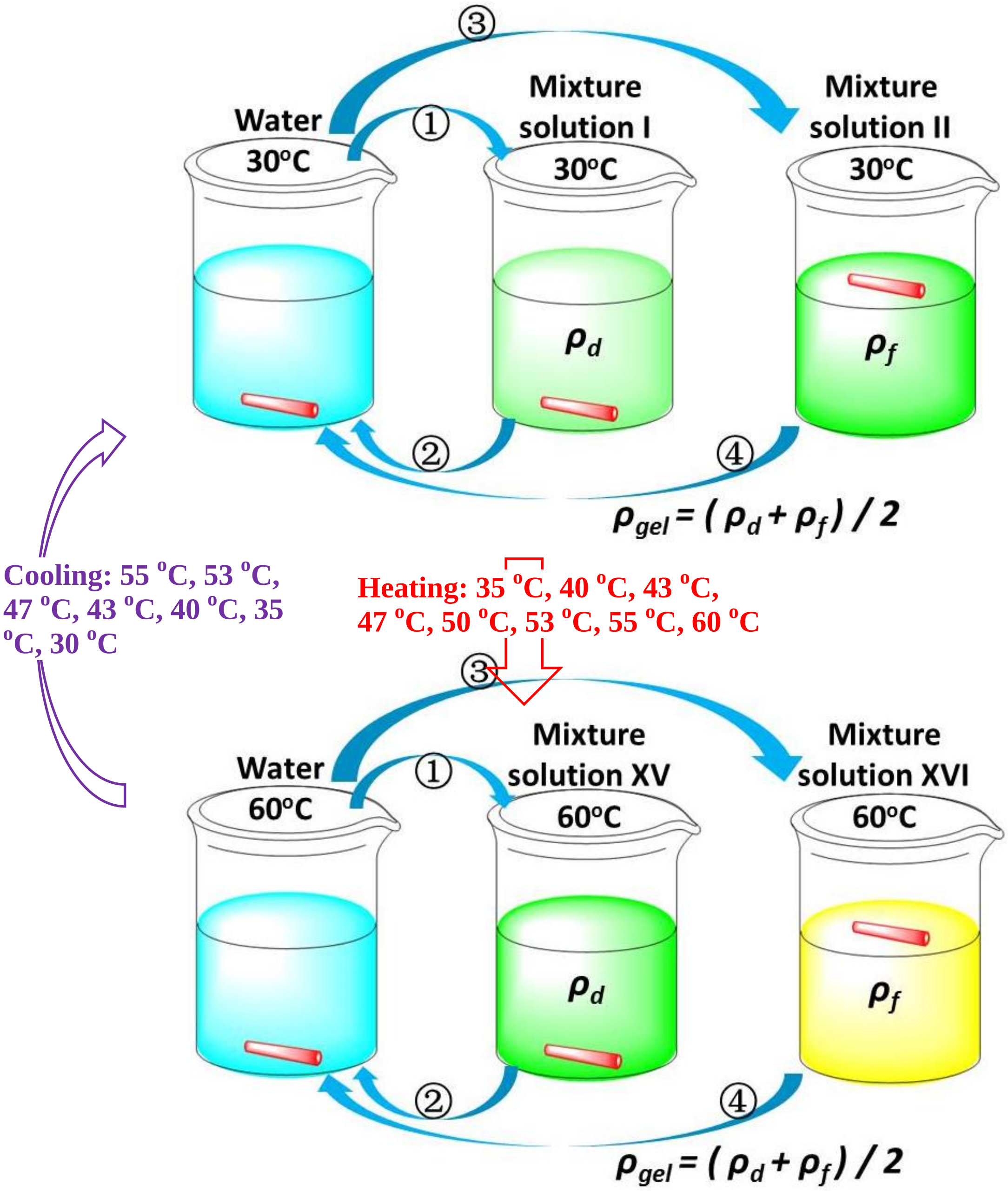

Figure S3. Density measurement of gels at different temperatures

Table S1. Density of glycerol solutions (g/cm^3)

Concentration (wt%)	Temperature (°C)								
	15[a]	20[a]	25[a]	30[a]	35[b]	40[b]	43[b]	47[b]	50[b]
1	1.00155	1.00060	0.99945	0.99800	0.99637	0.99444	0.99317	0.99313	0.98984
4	1.00875	1.00780	1.00655	1.00505	1.00326	1.00120	0.99983	0.99785	0.99626
9	1.02085	1.01970	1.01835	1.01670	1.01483	1.01270	1.01130	1.00930	1.00770

a. data from 'Handbook of physical properties of chemistry and chemical engineering: organic volume', ISBN: 7-5025-3760-0
b: calculated data after polynomial fitting the data from 15～30 °C, the polynomial fitting equations are as follows:
1 wt% glycerol solution: $\rho=1.00284+10^{-5}T+5\times10^{-6}T^2$, $R^2=0.99979$
4 wt% glycerol solution: $\rho=1.00998-5\times10^{-7}T+5.5\times10^{-6}T^2$, $R^2=0.99995$
9 wt% glycerol solution: $\rho=1.02274-5.1\times10^{-5}T+5\times10^{-6}T^2$, $R^2=0.99984$

Table S2. Density of ethanol solutions (g/cm^3)

Concen. (wt%)	Temperature (°C)								
	30[a]	35[a]	40[a]	43[b]	47[b]	50[b]	53[b]	55[b]	60[b]
1	0.99379	0.99217	0.99034	0.98908	0.98729	0.98585	0.98431	0.98324	0.98039
5	0.98670	0.98501	0.98311	0.98181	0.97996	0.97847	0.97689	0.97578	0.97285
9	0.98031	0.97846	0.97641	0.97503	0.97306	0.97148	0.96982	0.96865	0.96558
15	0.97133	0.96911	0.96670	0.96512	0.96291	0.96115	0.95932	0.95805	0.95473
27	0.95272	0.94955	0.94625	0.94420	0.94140	0.93923	0.93702	0.93552	0.93168
32	0.94370	0.94021	0.93662	0.93442	0.93142	0.92913	0.92681	0.92524	0.92124

a. data from 'Handbook of physical properties of chemistry and chemical engineering: organic volume', ISBN: 7-5025-3760-0
b: calculated data after polynomial fitting the data from 10～40 °C, the polynomial fitting equations are as follows:
1 wt% ethanol solution: $\rho=0.99842-6.21905\times10^{-6}T-4.90476\times10^{-6}T^2$, $R^2=0.99988$
5 wt% ethanol solution: $\rho=0.99163-1.39429\times10^{-5}T-4.98571\times10^{-6}T^2$, $R^2=0.99993$
9 wt% ethanol solution: $\rho=0.98625-4.93905\times10^{-5}T-4.91905\times10^{-6}T^2$, $R^2=0.99993$
15 wt% ethanol solution: $\rho=0.98002-1.56629\times10^{-4}T-4.41429\times10^{-6}T^2$, $R^2=0.99998$
27 wt% ethanol solution: $\rho=0.96893+4.59181\times10^{-4}T+2.69524\times10^{-6}T^2$, $R^2=1$
32 wt% ethanol solution: $\rho=0.96252-5.6658\times10^{-4}T-2.02381\times10^{-6}T^2$, $R^2=1$

Table S3. Density of pure water (g/cm^3)

Temperature (°C)									
30[a]	40[a]	42[a]	43[b]	44[a]	46[a]	47[b]	48[a]	50[a]	60[a]
0.99568	0.99225	0.99147	0.99107	0.99066	0.98982	0.99024	0.98896	0.98807	0.98324

a: data from 'Handbook of physical properties of chemistry and chemical engineering: inorganic volume', ISBN: 7-5025-3591-8
b: calculated data equal to the average of the two neighboring values, e.g. $\rho_{43\,^{o}C} = (\rho_{42\,^{o}C} + \rho_{44\,^{o}C}) / 2$

Table S4. Density of S53M47G0.6 at different temperatures

No.	Temperature (°C)	ρ_d (g/cm^3)	ρ_f (g/cm^3)	ρ_{gel} (g/cm^3) $\rho_{gel} = (\rho_d + \rho_f)/2$
1	30	1.00505 (4 wt% glycerol)	1.01670 (9 wt% glycerol)	1.01088
2	35	1.00326 (4 wt% glycerol)	1.01483 (9 wt% glycerol)	1.00905
3	40	1.00120 (4 wt% glycerol)	1.01270 (9 wt% glycerol)	1.00695
4	43	0.99983 (4 wt% glycerol)	1.01130 (9 wt% glycerol)	1.00556
5	47	0.98729 (1 wt% ethanol)	0.98939 (pure water)	0.98834
6	50	0.97847 (5 wt% ethanol)	0.98585 (1 wt% ethanol)	0.98219
7	53	0.97689 (5 wt% ethanol)	0.98431 (1 wt% ethanol)	0.98060
8	55	0.97578 (5 wt% ethanol)	0.98324 (1 wt% ethanol)	0.97951
9	60	0.97285 (5 wt% ethanol)	0.98039 (1 wt% ethanol)	0.97662
10	55	0.97578 (5 wt% ethanol)	0.98324 (1 wt% ethanol)	0.97951
11	53	0.97689 (5 wt% ethanol)	0.98431 (1 wt% ethanol)	0.98060
12	50	0.97847 (5 wt% ethanol)	0.98585 (1 wt% ethanol)	0.98219
13	47	0.97996 (5 wt% ethanol)	0.98729 (1 wt% ethanol)	0.98363
14	43	0.99107 (pure water)	0.99317 (1 wt% glycerol)	0.99212
15	40	0.99444 (1 wt% glycerol)	1.0012 (4 wt% glycerol)	0.99782
16	35	1.00326 (4 wt% glycerol)	1.01483 (9 wt% glycerol)	1.00905
17	30	1.00505 (4 wt% glycerol)	1.01670 (9 wt% glycerol)	1.01088

ρ_d (the density of the solution, where the gel rod dives)
ρ_f (the density of the solution, where the gel rod floats)
ρ_{gel} (the density of the gel rod calculated via ρ_d.and ρ_f ; $\rho_{gel} = (\rho_d + \rho_f)/2$)

Based on the density of monomers (0.8 g/cm^3 for SA, 1.0153 g/cm^3 for MA), the calculated density of S53M47G0.6 is 0.9 g/cm^3, different from the measured one (1.01088 g/cm^3). There is a volume contraction during polymerization, which is well-known in polymer science. So the volume of obtained polymers is lower than that of the corresponding monomers, which gives rise to an increase in density, e.g. the density of methacrylic acid and poly(methacrylic acid) are 1.0153g/cm^3 and 1.285 g/cm^3 (Table 1.15 Physical constants of organic compounds. Section 1. Organic compounds, J. A. Dean, *Lange's handbook of chemistry*, 15th ed., McGRAW-HILL, INC, 1998.; James E. Mark, *Polymer Data Handbook*, Oxford University Press, 1998, 638), respectively. This leads to the deviation.

The reasons for choosing these compositions (S53M47G0.6 and S82M18G0.6) are as follows.

Water does not absorb NIR laser to increase its temperature. Compared with the density of water at 30°C (0.99568g/cm^3), the density of S53M47G0.6 is 1.5% higher at 30°C (1.01088 g/cm^3), and 1.5% lower at 60°C (0.98039 g/cm^3). This means that both floating and diving velocities of S53M47G0.6 is fast. If the content of SA is lower than 53 mol%, the floating rate is low, and the diving rate is high, and vice versa. In the case of S82M18G0.6, the content of SA is very high, so it is very sticky as temperature is above T_m. This makes it convenient to fabricate composite mini-robot by sticking S82M18G0.6 and S53M47G0.6. The density of S82M18G0.6 is lower than that of water, which makes it possible to realize a stable 'stand up' pose during the 'bipedal walking' motion. In addition, the obtained samples are too brittle to use at room temperature when the content of SA is higher than 82 mol%. Therefore, we chose S53M47G0.6 and S82M18G0.6.

Table S5. Density of S82M18G0.6 at different temperatures

No.	Temperature (°C)	ρ_d (g/cm^3)	ρ_f (g/cm^3)	ρ_{gel} (g/cm^3) $\rho_g = (\rho_d + \rho_f)/2$
1	30	0.98031 (9 wt% ethanol)	0.98670 (5 wt% ethano l)	0.98351
2	35	0.97846 (9 wt% ethanol)	0.98501 (5 wt% ethanol)	0.98174
3	40	0.97641 (9 wt% ethanol)	0.98311 (5 wt% ethanol)	0.97976
4	43	0.97503 (9 wt% ethanol)	0.98181 (5 wt% ethanol)	0.97842
5	47	0.96291 (15 wt% ethanol)	0.97306 (9 wt% ethanol)	0.96798
6	50	0.92913 (32 wt% ethanol)	0.93923 (27 wt% ethanol)	0.93418
7	53	0.92681 (32 wt% ethanol)	0.93702 (27 wt% ethanol)	0.93191
8	55	0.92524 (32 wt% ethanol)	0.93552 (27 wt% ethanol)	0.93038
9	60	0.92124 (32 wt% ethanol)	0.93168 (27 wt% ethanol)	0.92646
10	55	0.92524 (32 wt% ethanol)	0.93552 (27 wt% ethanol)	0.93038
11	53	0.92681 (32 wt% ethanol)	0.93702 (27 wt% ethanol)	0.93191
12	50	0.92913 (32 wt% ethanol)	0.93923 (27 wt% ethanol)	0.93418
13	47	0.93142 (32 wt% ethanol)	0.94140 (27 wt% ethanol)	0.93641
14	43	0.96512 (15 wt% ethanol)	0.97503 (9 wt% ethanol)	0.97008
15	40	0.97641 (9 wt% ethanol)	0.98311 (5 wt% ethanol)	0.97976
16	35	0.97846 (9 wt% ethanol)	0.98501 (5 wt% ethanol)	0.98174
17	30	0.98031 (9 wt% ethanol)	0.98670 (5 wt% ethano l)	0.98351

ρ_d (the density of the solution, where the gel rod dives)
ρ_f (the density of the solution, where the gel rod floats)
ρ_{gel} (the density of the gel rod calculated via ρ_d.and ρ_f; $\rho_{gel} = (\rho_d + \rho_f)/2$)

The magnitude of the force generated to achieve the motions can be approximately speculated in terms of the density of water and hydrogels.

In the case of floating/diving motions of S53M47GO0.6 rod, (φ=1.8 mm, length=12 mm)

ρ_{gel} at 30 °C =1.01088 g/cm^3,

V_{rod} = 3.14*(0.9 mm)^2*12 mm=30.52 mm^3 = 30.52*10^{-3} cm^3,

$F_c = G - F_b = \rho_{gel} * V_{rod} * g - \rho_{water} * V_{rod} * g$

$= (\rho_{gel} - \rho_{water}) * V_{rod} * g$

$= (1.01088 - 0.99568\ g/cm^3) * 30.52*10^{-3}\ cm^3 * 9.8\ mN/g$

$= 0.0152\ g/cm^3 * 30.52*10^{-3}\ cm^3 * 9.8\ mN/g$
$= 4.546 *10^{-3}\ mN$
$= 4.546\ \mu N$

As the increasing buoyancy resulting from the NIR laser is higher than 4.546 μN, the hydrogel rod will exhibit a floating motion.

Somersaulting motion of S53M47GO0.6 rectangular gel plate, (0.9×4.8×12.7 mm)
ρ_{gel} at 30°C =1.01088g/cm^3,
$V_{rec.} = 0.9\ mm * 4.8\ mm * 12.7\ mm = 54.86\ mm^3 = 54.86 * 10^{-3}\ cm^3$,
$F_c = G - F_b = \rho_{gel} * (V_{rec.}) * g - \rho_{water} * V_{rec.} * g = (\rho_{gel} - \rho_{water}) * V_{rec.} * g$
$= (1.01088 - 0.99568\ g/cm^3) * 54.86 * 10^{-3}\ cm^3 * 9.8\ mN/g$
$= 0.0152\ g/cm^3 * 54.86*10^{-3}\ cm^3 * 9.8\ mN/g$
$= 8.172 *10^{-3}\ mN$
$= 8.172\ \mu N$.

Based on torque balance, the required composite force for somersaulting motion $F'_c = F_c*(L/2)/L = F_c/2 = 8.172/2\ \mu N = 4.086\mu N$.

Rolling motion: Theoretically, any slightly increasing buoyancy in one side of the thick gel rod will break the torque balance, leading to a rolling motion.

Thus, the force generated to achieve the motions is in the magnitude of μN.
